# Xyloketal B Suppresses Glioblastoma Cell Proliferation and Migration *in Vitro* through Inhibiting TRPM7-Regulated PI3K/Akt and MEK/ERK Signaling Pathways

**DOI:** 10.3390/md13042505

**Published:** 2015-04-22

**Authors:** Wen-Liang Chen, Ekaterina Turlova, Christopher L. F. Sun, Ji-Sun Kim, Sammen Huang, Xiao Zhong, Yong-Yuan Guan, Guan-Lei Wang, James T. Rutka, Zhong-Ping Feng, Hong-Shuo Sun

**Affiliations:** 1 Department of Physiology, Faculty of Medicine, University of Toronto, Toronto, ON M5S 1A8, Canada; E-Mails: wenliang.chen@utoronto.ca (W.-L.C.); e.turlova@mail.utoronto.ca (E.T.); jsk.kim@mail.utoronto.ca (J.-S.K.); sammen.huang@mail.utoronto.ca (S.H.); 16822832@qq.com (X.Z.); 2 Department of Surgery, Faculty of Medicine, University of Toronto, Toronto, ON M5S 1A8, Canada; E-Mail: james.rutka@sickkids.ca; 3 Department of Pharmacology, Faculty of Medicine, University of Toronto, Toronto, ON M5S 1A8, Canada; 4 Faculty of Applied Science & Engineering, University of Toronto, Toronto, ON M5S 1A4, Canada; E-Mail: christopher.sun@mail.utoronto.ca; 5 Department of Pharmacology, Zhongshan School of Medicine, Sun Yat-sen University, Guangzhou 510080, China; E-Mails: guanyy@mail.sysu.edu.cn (Y.-Y.G.); wangglei@mail.sysu.edu.cn (G.-L.W.); 6 Key Laboratory of Functional Molecules from Oceanic Microorganisms, Department of Education of Guangdong Province, Sun Yat-sen University, Guangzhou 510080, China; 7 Institute of Medical Science, Faculty of Medicine, University of Toronto, Toronto, ON M5S 1A8, Canada

**Keywords:** glioblastoma, xyloketal B, proliferation, migration, TRPM7, marine compound

## Abstract

Glioblastoma, the most common and aggressive type of brain tumors, has devastatingly proliferative and invasive characteristics. The need for finding a novel and specific drug target is urgent as the current approaches have limited therapeutic effects in treating glioblastoma. Xyloketal B is a marine compound obtained from mangrove fungus *Xylaria* sp. (No. 2508) from the South China Sea, and has displayed antioxidant activity and protective effects on endothelial and neuronal oxidative injuries. In this study, we used a glioblastoma U251 cell line to (1) explore the effects of xyloketal B on cell viability, proliferation, and migration; and (2) investigate the underlying molecular mechanisms and signaling pathways. MTT assay, colony formation, wound healing, western blot, and patch clamp techniques were employed. We found that xyloketal B reduced cell viability, proliferation, and migration of U251 cells. In addition, xyloketal B decreased p-Akt and p-ERK1/2 protein expressions. Furthermore, xyloketal B blocked TRPM7 currents in HEK-293 cells overexpressing TRPM7. These effects were confirmed by using a TRPM7 inhibitor, carvacrol, in a parallel experiment. Our findings indicate that TRPM7-regulated PI3K/Akt and MEK/ERK signaling is involved in anti-proliferation and migration effects of xyloketal B on U251 cells, providing *in vitro* evidence for the marine compound xyloketal B to be a potential drug for treating glioblastoma.

## 1. Introduction

Glioblastoma Multiforme (GBM) is the highest grade glioma (grade IV) tumor and the most malignant form of astrocytoma. Despite the wide range of treatments, including surgery, radiotherapy, and chemotherapy, a majority of the therapies for GBM have a limited improvement on patients’ survival, largely due to the highly proliferative, invasive, and often drug-resistant nature of the tumor. GBM’s median survival time is approximately 14.6 months [1]. Because of the ineffective outcomes with conventional therapies, finding novel and specific drug targets for GBM is still a challenge.

Proliferation, survival, and motility of glioblastoma cells are regulated by different intracellular signaling pathways. Among these, the Ras/MAP kinase-ERK kinase (MEK)/extracellular-signal-regulated kinase (ERK) pathway and PI3K/Akt pathway have long been established. A large number of genetic abnormalities were uncovered in human glioblastoma samples, and the most prominent one is deregulation of signal transduction pathways [2]. It happens in glioblastoma through upregulation or a gain-of-function mutation of receptor tyrosine kinases (RTKs), such as epidermal growth factor receptor (EGFR), platelet-derived growth factor receptor (PDGFR), and fibroblast growth factor receptor (FGFR) [3,4]. These abnormalities cause constitutive activation of Ras/MEK/ERK, PI3K/Akt, and other signal transduction pathways [5]. Novel treatments targeting RTKs, PI3K/Akt, and MEK/ERK signaling pathway are currently under evaluation in clinical trials [6]. However, the majority of glioblastoma patients fail to respond to treatments with either PI3K/Akt or MEK/ERK signaling inhibitors [7], suggesting that a single suppression of one signaling pathway may be insufficient for effectively treating this tumor. Therefore, strategies that combine blockage of these two signaling pathways may be essential for the successful glioblastoma treatment. Further study is required to find the novel target in the upstream of these two signaling pathways.

Accumulating studies raise the notion that ion channels play critical roles in the malignant behavior of glioblastoma cells. Thus it would be possible to regulate certain ion channels in glioblastoma cells in order to suppress tumor cell proliferation, migration, and invasion. Several ion channels are involved in regulating the behavior of glioblastoma cells, such as ClC-3, K_ATP_, and TRPM7 channels [8,9,10,11]. TRPM7 channel, a calcium-conducting divalent cation channel, is a member 7 of the Melastatin subfamily of the Transient Receptor Potential ion channel superfamily, and is ubiquitously expressed in almost all tissues. TRPM7 plays a vital role in embryonic development, anoxia/ischemia, cardiovascular disease, and cancer [12,13,14]. TRPM7 overexpression was found in tissues of several cancer types [15,16]. Recent reports have shown that TRPM7 controls proliferation, migration, and invasion of glioblastoma cells and glioma stem cells [3,10], suggesting that TRPM7 could potentially serve as a clinical biomarker and therapeutic target for glioblastoma [17].

**Figure 1:**
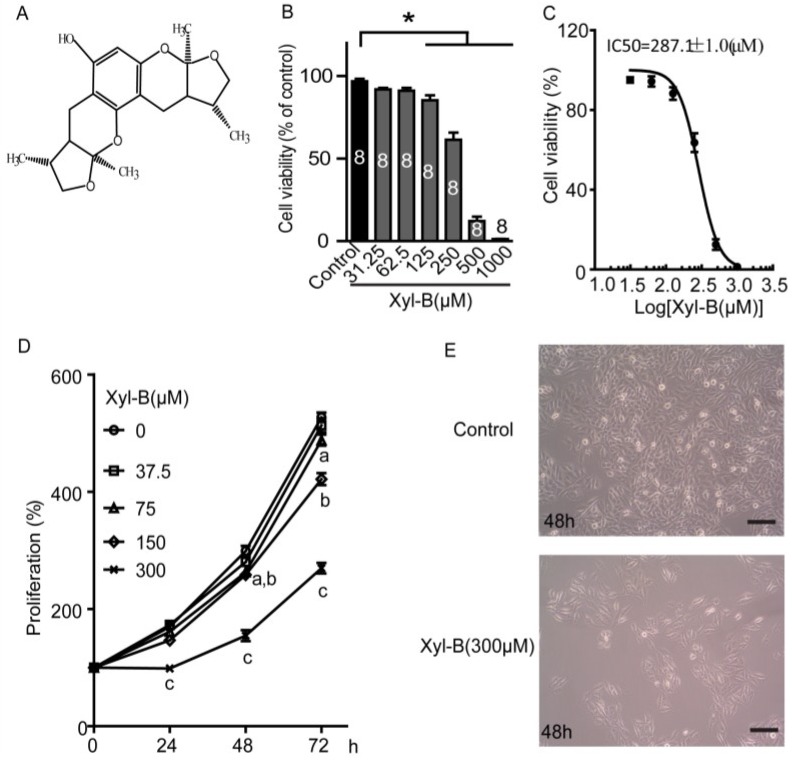

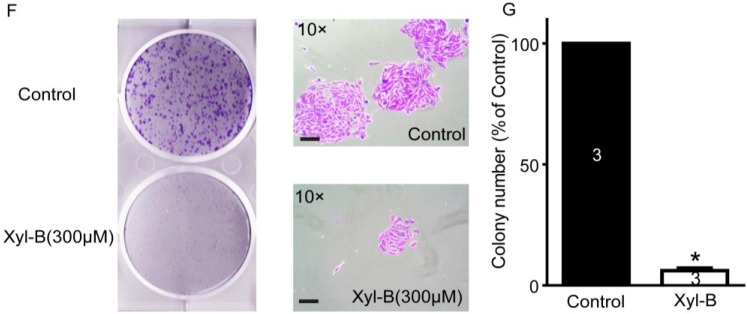
Effects of xyloketal B (Xyl-B) on cell viability and proliferation of U251 cells. (**A**) Chemical structure of xyloketal B; (**B**) Xyloketal B concentration-dependently reduced the cell viability of U251 cell line. U251 cells were incubated with xyloketal B (31.25–1000 μM) for 24 h, following MTT assay. * *p* < 0.05, *n* = 8 independent experiments; (**C**) Nonlinear curve fit for dose-response of xyloketal B treatment in U251 cells for 24 h. IC_50_ = 287.1 ± 1.0 μM; (**D**) Xyloketal B inhibited proliferation of U251 cell line. U251 cells were treated with xyloketal B for 24, 48, and 72 h, and then cell proliferation was detected by MTT assay; a, b, and c represent 75, 150, and 300 μm xyloketal B *versus* the control group, respectively, *p* < 0.05, *n* = 6 independent experiments; (**E**) Representative images of U251 cells with or without xyloketal B treatment for 48 h showed reduction of cell numbers in xyloketal B treatment group. Cell images were obtained with a digital camera connected to a phase-contrast Olympus microscope (CKX41, ×10 objectives). *n* = 3; (**F**) Xyloketal B inhibited colony formation of U251 cells. Cells were plated in six-well culture plates and treated with xyloketal B (300 μM) for 24 h. The culture medium was changed at regular time intervals. Colony formation of U251 cells was detected by crystal violet staining at seven days after xyloketal B treatment. Images were taken using a scanner (CanoScan LiDE 700F, left panel) and a digital camera connected to a phase-contrast Olympus microscope (CKX41, ×10 objectives, right panel). Colony numbers were calculated using Image-Pro Plus software. Representative images were shown. *n* = 3; (**G**) Statistic analysis of colony formation results. Xyloketal B significantly reduced the colony formation of the U251 cells. * *p* < 0.05, *n* = 3. All scale bars = 150 μm.

Drug discovery from marine organisms began 60 years ago. There are some successful examples of discovering, developing, and introducing clinical agents derived from marine sources, including the analgesic ziconotide and the anti-cancer compound trabectedin [18,19]. Xyloketal B (chemical structure of xyloketal B shown in Figure 1A) is a novel marine compound isolated from mangrove fungus *Xylaria* sp. (No. 2508) from the South China Sea [20]. Xyloketal B has displayed several bioactive effects, such as protective effects against oxidative endothelial injury, alleviating oxygen glucose deprivation (OGD)-induced mitochondria dysfunction and injury in PC12 cells, protecting against MPP+-induced neurotoxicity in *C. elegans* and PC12 cells, antioxidant activity in endothelial cell and zebrafish through regulating HO-1, and reducing hypoxia-ischemia-induced brain injury of neonatal mice [21,22,23,24,25]. Our preliminary study indicated that xyloketal B reduced cell viability of glioblastoma U251 cells in a dose-dependent manner. This study further reveals the effects of xyloketal B on cell proliferation and migration of U251 cells and its underlying signaling pathway.

## 2. Results and Discussion

### 2.1. Xyloketal B Reduces U251 Cell Viability

Firstly, the effects of xyloketal B on cell viability were assessed using MTT assay [21]. As shown in Figure 1B, various concentrations of xyloketal B (from 31.25 to 1000 μM) treatment for 24 h reduced U251 cell viability in a concentration-dependent manner. The cell viability significantly decreased to 85.4% ± 2.9%, 61.4% ± 4.3%, 12.2% ± 2.6% and 1.3% ± 0.1% of control in 125 μM, 250 μM, 500 μM, and 1000 μM xyloketal B, respectively (* *p* < 0.05, *n* = 8). Nonlinear curve fit was carried out to evaluate the dose-response of xyloketal B, and the IC_50_ of xyloketal B was equal to 287.1 ± 1.0 μM (Figure 1C). The concentrations of xyloketal B used in the following experiments were chosen according to this IC_50_ value.

### 2.2. Xyloketal B Inhibits U251 Cell Proliferation

Next, cell proliferation was detected using MTT assay [21]. The number of living cells is proportional to the OD value of MTT assay. U251 cells were incubated with 37.5–300 μM xyloketal B for 24, 48, and 72 h before MTT assay was carried out. The OD values of MTT assay were detected once U251 cells were treated with various concentration of xyloketal B and set as a baseline of cell proliferation (100%). As shown in Figure 1D, xyloketal B treatment for 24 h inhibited U251 cell proliferation at 300 μM, showing 98.5% ± 5.9% of baseline in xyloketal B (300 μM) and 169.4% ± 1.9% of baseline in control group (*p* < 0.05, *n* = 6). When U251 cells were incubated with xyloketal B for 48 and 72 h, cell proliferation was significantly inhibited by xyloketal B at lower concentrations up to 75 μM (*p* < 0.05, *n* = 6). These data indicate that the inhibitive effects of xyloketal B on cell proliferation are time- and concentration-dependent. In addition, xyloketal B (<300 μM) mainly displayed inhibition of cell proliferation, rather than producing cytotoxic effects on the U251 cells. Cell images were also taken at 48 h after treatment with xyloketal B (300 μM), and showed no significant cell damage, but displayed a decreasing cell density compared to the control group, which had a higher cell density in the visual field (Figure 1E, *n* = 3). *In vitro* colony formation assay, which is a cell survival assay, evaluates the ability of a single cell to grow into a colony and also is used to assess the long-term effects on cell proliferation [26]. As shown in Figure 1F,G, a large number of U251 cell colonies was seen in the control group after seeding in six-well plates for seven days with crystal violet staining. The colony formation of U251 cells was significantly decreased after the xyloketal B (300 μM) treatment to 6.1% ± 1.1% of the control (* *p* < 0.05, *n* = 3). The results of colony formation assay further demonstrated the inhibitive effects of xyloketal B on U251 cell proliferation.

### 2.3. Xyloketal B Inhibits U251 Cell Migration

**Figure 2:**
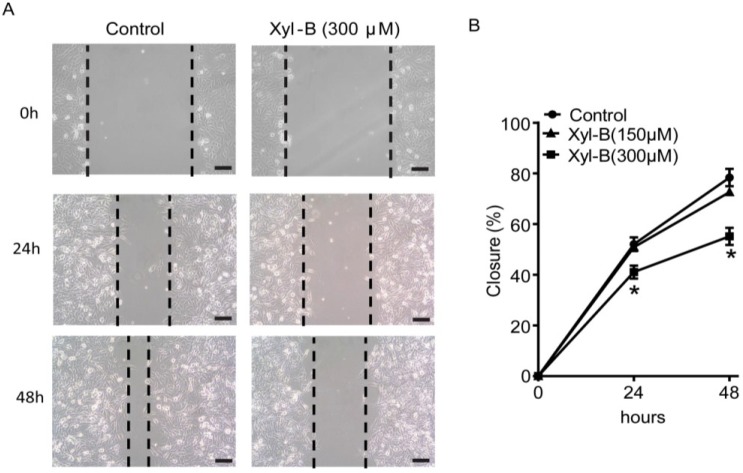
Effects of xyloketal B on the migration of U251 cells. (**A**) Xyloketal B inhibited U251 cell migration. The representative images of wound healing assay were displayed. After being scratched with a 200-μL pipette tip, U251 cells were treated with xyloketal B (300 μM) or vehicle (0.1% DMSO), then images were taken at 0, 24, and 48 h, and gap closure was analyzed; (**B**) Statistical analysis of migration results. Xyloketal B significantly inhibited the cell migration of the U251 cells in both timelines tested. * *p* < 0.05, *n* = 3. Scale bars = 150 μM.

Wound healing assay was used to evaluate the cell migration [27]. As shown in Figure 2, xyloketal B (300 μM) treatment significantly inhibited U251 cell migration (* *p* < 0.05, *n* = 3). The gap closures in xyloketal B (300 μM) group were 41.1% ± 2.6% and 55.1% ± 3.4%, compared to 52.1% ± 2.7% and 78.4% ± 3.4% in the control group at 24 and 48 h, respectively.

### 2.4. Xyloketal B Suppresses the PI3K/Akt and MEK/ERK Signaling Pathways

**Figure 3:**
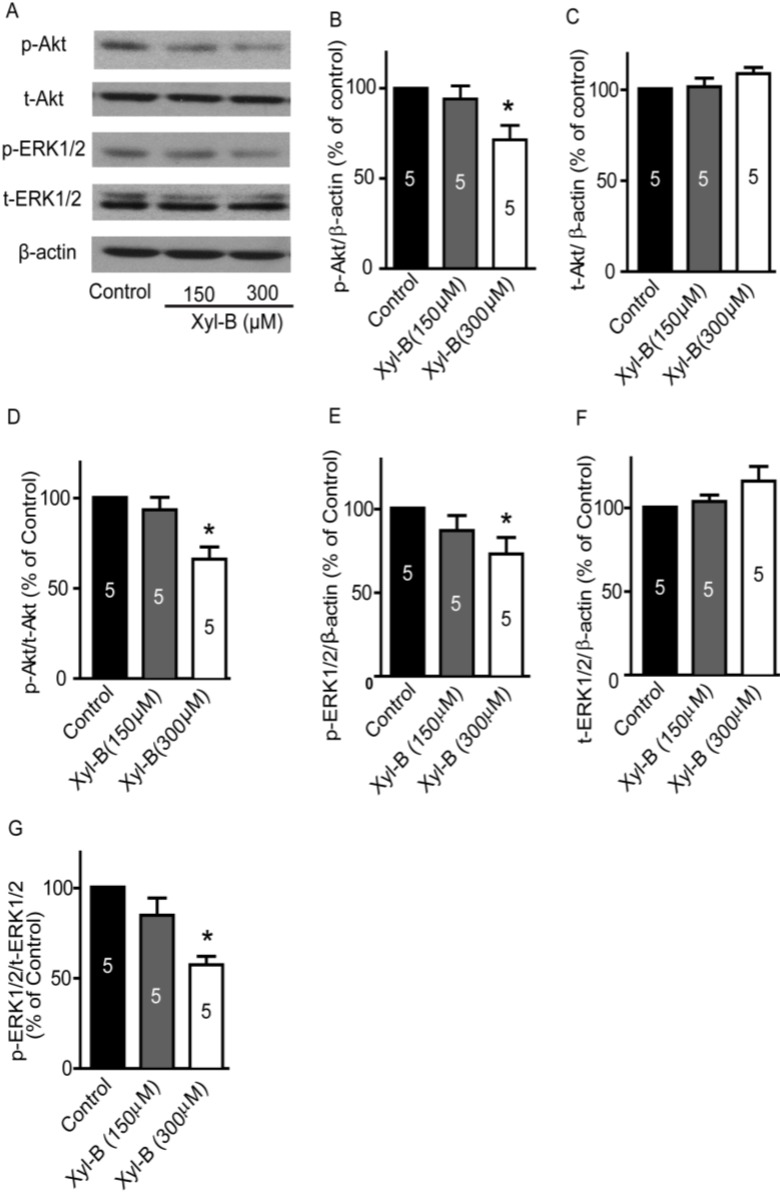
Effects of xyloketal B on p-ERK, t-ERK, p-Akt, and t-Akt protein expressions. U251 cells were treated with xyloketal B (150 and 300 μM) for 24 h, and then the protein expression profile was detected by western blots. (**A**) Representative images of western blotting results; (**B**) Xyloketal B (300 μM) treatment significantly reduced p-Akt protein expression. * *p* < 0.05, *n* = 5; (**C**) Xyloketal B did not significantly alter the t-Akt protein expression; (**D**) Ratio of p-Akt/t-Akt decreased in the xyloketal B (300 μM) treatment group. * *p* < 0.05, *n* = 5; (**E**) Xyloketal B (300 μM) treatment significantly reduced p-ERK1/2 protein expression. * *p* < 0.05, *n* = 5; (**F**) Xyloketal B did not significantly alter t-ERK1/2 protein expression; (**G**) Ratio of p-ERK1/2/t-ERK1/2 decreased in xyloketal B (300 μM) treatment group. * *p* < 0.05, *n* = 5.

PI3K/Akt and MEK/ERK signaling pathways are involved in the regulation of proliferation and migration of glioblastoma cells [28]. At present, therapeutic agents targeting both signaling pathways have been developed for treating recurrent malignant glioma patients, and are currently in clinical trials [29]. In order to evaluate the underlying mechanisms of xyloketal B regulating U251 cell proliferation and migration, western blotting was carried out to detect alterations in p-Akt/t-Akt and p-ERK1/2/t-ERK1/2, which are key signaling proteins of PI3K/Akt and MEK/ERK signaling pathways. As shown in Figure 3A, the representative images of western blotting showed that both p-Akt and p-ERK1/2 protein expressions in U251 cells were significantly reduced by xyloketal B (300 μM) treatment for 24 h. Densitometry analysis indicated that p-Akt protein expression, normalized to β-actin, decreased in the xyloketal B (300 μM) treatment group (Figure 3B, 71.5% ± 8.0% of control, * *p* < 0.05, *n* = 5), while the total Akt (t-Akt) protein expression did not change significantly (Figure 3C, 108.4% ± 3.4% of control, *p* > 0.05, *n* = 5). The ratio of p-Akt/t-Akt decreased in the xyloketal B (300 μM) group (Figure 3D, 65.9% ± 6.8% of control, * *p* < 0.05, *n* = 5). In addition, xyloketal B (300 μM) treatment for 24 h reduced p-ERK1/2 protein expression and p-ERK1/2-t-ERK1/2 ratio in U251 cells (Figure 3E,G, p-ERK1/2/β-actin: 72.6% ± 9.9% of control; p-ERK1/2/t-ERK1/2: 60.0 ± 4.7% of control, respectively; * *p* < 0.05, *n* = 5). The total ERK1/2 protein expression did not change significantly (Figure 3F, *p* > 0.05, *n* = 5).

### 2.5. Xyloketal B Blocks the TRPM7 Current

**Figure 4:**
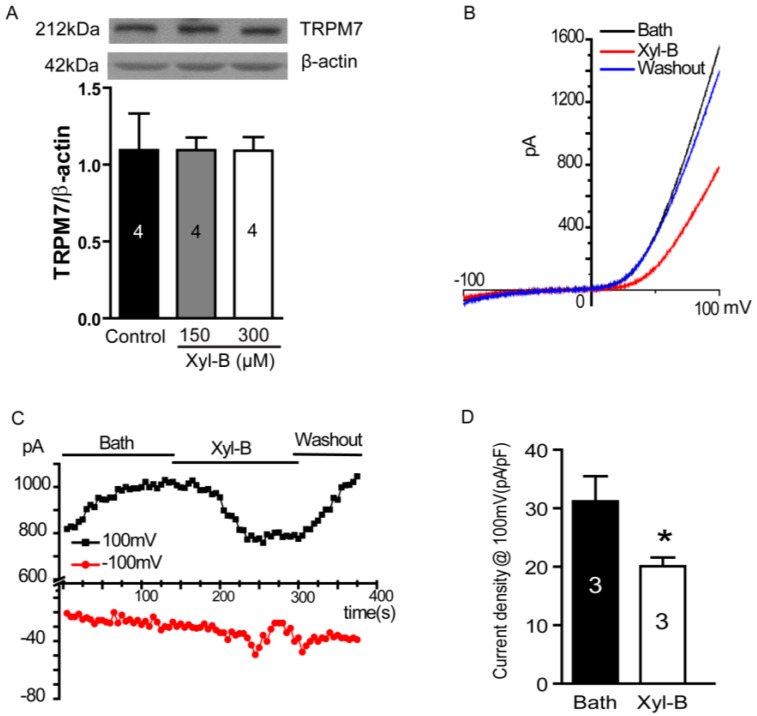
Effects of xyloketal B on TRPM7 currents in HEK-293 cell over-expressing TRPM7. (**A**) Xyloketal B did not significantly regulate TRPM7 protein expressions in U251 cells. U251 cells were treated with xyloketal B (150 and 300 μM) for 24 h followed by detection with western blotting. *n* = 4; (**B**) Xyloketal B (300 μM) blocked TRPM7 currents. TRPM7 protein overexpression in HEK-293 cells was induced by treatment with tetracycline (Tet, 1 μg/mL) for 24 h. Then, TRPM7 currents were recorded using the whole-cell patch-clamp technology with ramp from −100 mV to 100 mV. Representative I–V traces were shown. *n* = 3; (**C**) Representative time course of the inward and outward current of TRPM7 at −100 and 100 mV. *n* = 3; (**D**) Statistical analysis of patch-clamp experiments. Xyloketal B (300 μM) perfusion significantly reduced the outward current of TRPM7. * *p* < 0.05, *n* = 3.

The above results show that the PI3K/Akt and MEK/ERK signaling pathways are regulated by xyloketal B. We further explored the upstream signaling protein for both signaling pathways. Recently, Leng *et al.* reported that the suppression of TRPM7 reduced the proliferation, migration, and invasion of A172 cells, a human glioma cell line [10]. In hepatic stellate cells, TRPM7 regulates its proliferation via the PI3K and ERK pathways [30]. Therefore, we next explored whether xyloketal B could regulate TRPM7 and thus the PI3K/Akt and MEK/ERK signaling pathways. First, we carried out western blotting experiments to detect the TRPM7 protein expression in U251 cells and the effects of xyloketal B on TRPM7 expression. As shown in Figure 4A, TRPM7 protein was found to be expressed in U251 cells. Xyloketal B (150 and 300 μM) did not significantly regulate TRPM7 protein expression (*p* > 0.05, *n* = 4). Next, we performed whole-cell patch-clamp experiments to test the effects of xyloketal B on the TRPM7 current in HEK-293 cells overexpressing TRPM7. As shown in Figure 4B–D, xyloketal B (300 μM) perfusion blocked the TRPM7 current; its inhibitory effect was eliminated by washout of a bath solution, suggesting it was specific and reversible. The inhibitory efficiency of xyloketal B at 300 μM was approximately 33.4% (Figure 4D, * *p* < 0.05, *n* = 3).

### 2.6. TRPM7 Inhibitor Carvacrol Reduces U251 Cell Viability, Proliferation, and Migration

**Figure 5:**
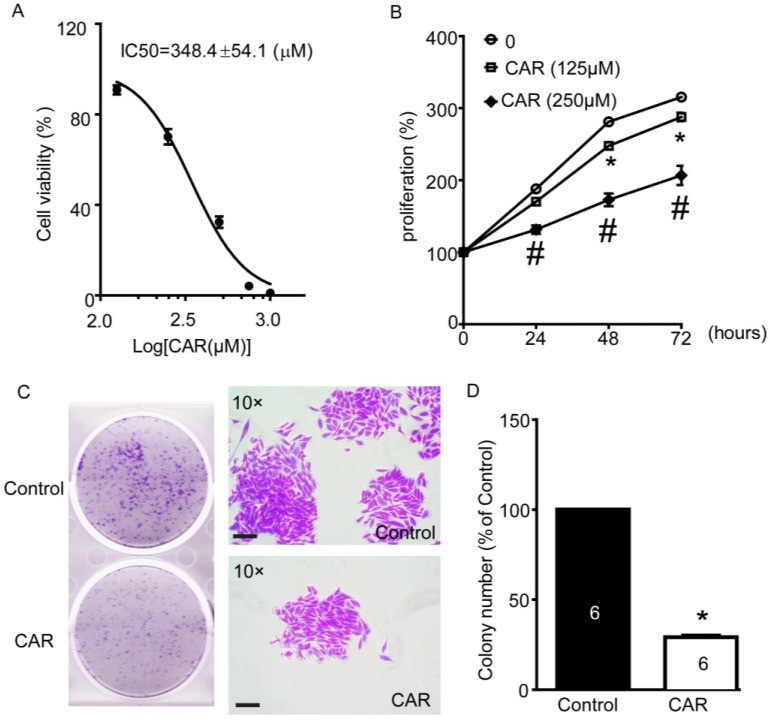

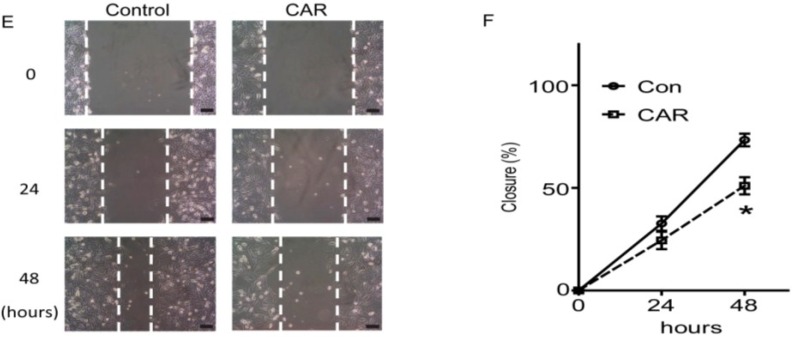
Effects of TRPM7 inhibitor carvacrol (CAR) on viability, proliferation, and migration of U251 cells. (**A**) Carvacrol reduced the viability of U251 cells. U251 cells were treated with various concentrations of carvacrol for 24 h following detection with MTT assay. Nonlinear curve fitting for dose-response of carvacrol treatment was displayed and IC_50_ was calculated as 348.4 ± 54.1 μM. *n* = 8 independent experiments; (**B**) Carvacrol inhibited the proliferation of U251 cells. U251 cells were treated with carvacrol (125 and 250 μM) or vehicle control (0.1% DMSO) for 24, 48, or 72 h. Cell proliferation was measured using MTT assay. * *p* < 0.05, carvacrol (125 μM) group *versus* control group; # *p* < 0.05, cavarcrol (250 μM) group *versus* control group, *n* = 8 independent experiments; (**C**) Carvacrol inhibited colony formation of U251 cells. Cells were plated in six-well culture plates and treated with carvacrol (500 μM) for 24 h. Colony formation of U251 cells was detected by crystal violet staining at seven days after carvacrol treatment. Images were taken using a scanner (CanoScan LiDE 700F, left panel) and a digital camera connected to a phase-contrast Olympus microscope (CKX41, ×10 objectives, right panel). Colony numbers were calculated using Image-Pro Plus software. Representative images were shown. *n* = 6; (**D**) Statistic analysis of colony formation results. Carvacrol B significantly reduced the colony formation of the U251 cells. * *p* < 0.05, *n* = 6; (**E**) Carvacrol inhibited U251 cell migration. The representative images of wound healing were displayed. After being scratched with 200-μL pipette tip, U251 cells were treated with carvacrol (500 μM) or vehicle (0.1% DMSO), then images were taken at 0, 24, and 48 h and gap closure was analyzed; (**F**) Statistical analysis of migration results. Carvacrol significantly inhibited the cell migration of the U251 cells at 48 h timeline. * *p* < 0.05, *n* = 4. All scale bars = 150 μM.

Carvacrol, a naturally synthesized, bioactive monoterpenoid phenol, was reported to block TRPM7 currents in HEK cells heterologously expressing mammalian TRPM7 and ectopically expressed in a primary culture of CA3-CA1 hippocampal brain neurons [31]. In our previous study, we have also confirmed blocking of TRPM7 currents in HEK-293 cells overexpressing TRPM7 (data not shown). Hence, we further applied carvacrol as a TRPM7 inhibitor to study the role of TRPM7 in U251 cell functions and underlying signaling pathways, to support the results of xyloketal B. As shown in Figure 5A, we found that 24 h treatment of carvacrol decreased U251 cell viability in a dose-dependent manner, while its IC_50_ was 348.4 ± 54.1 μM. Carvacrol at 150 and 250 μM inhibited U251 cell proliferation (Figure 5B). Carvacrol (150 and 250 μM) treatment for 72 h decreased cell proliferation by 206.6% ± 13.4% of baseline and 287.7% ± 6.0% of baseline, respectively, compared to 315.2% ± 5.3% of baseline in the control group (Figure 5B, * # *p* < 0.05, *versus* control group, *n* = 8). In addition, carvacrol (500 μM) incubation significantly reduced colony formation of U251 cells (Figure 5C,D, 28.9% ± 1.0% of control, * *p* < 0.05, *n* = 6). We also detected the effects of carvacrol on cell migration in the wound-healing assay. As shown in Figure 5E,F, we found that carvacrol (500 μM) significantly reduced U251 cell migration to approximately 30% of control (* *p* < 0.05, *n* = 4).

### 2.7. TRPM7 Inhibitor Carvacrol Suppresses the PI3K/Akt and MEK/ERK Signaling Pathways

**Figure 6:**
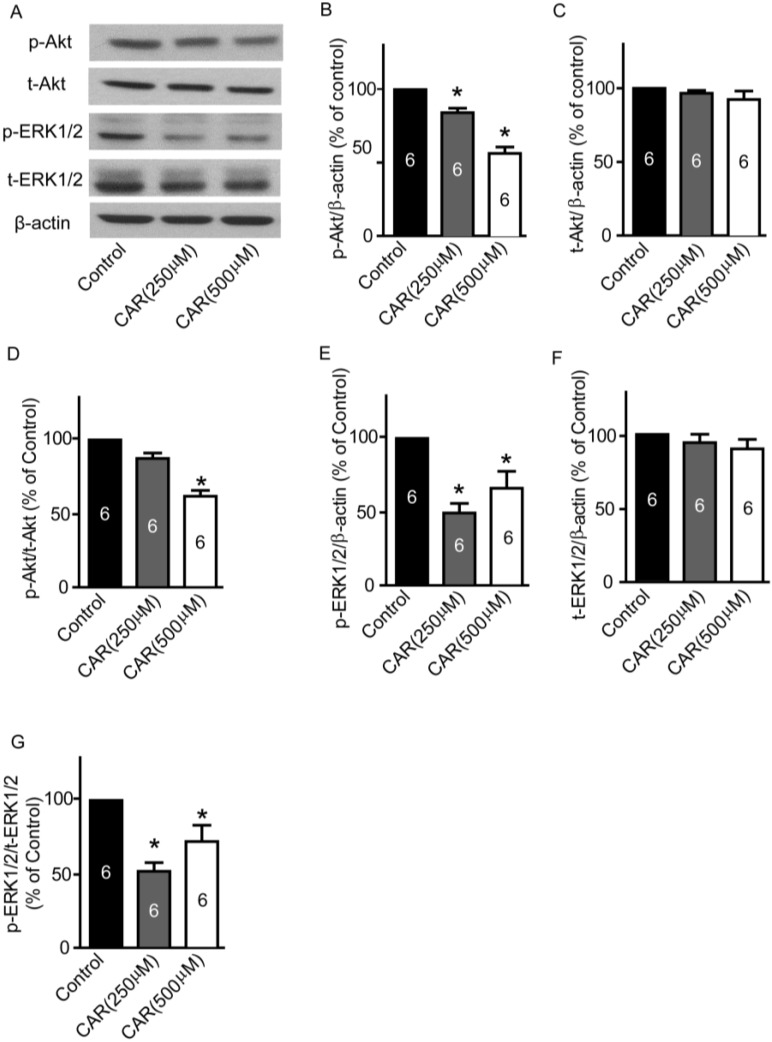
Effects of carvacrol (CAR) on p-ERK, t-ERK, p-Akt, and t-Akt protein expressions. U251 cells were treated with carvacrol (250 and 500 μM) for 24 h, and then protein expressions were detected by western blotting. (**A**) Representative images of western blotting results; (**B**) Carvacrol (500 μM) treatment significantly reduced p-Akt protein expression. * *p* < 0.05, *n* = 6; (**C**) Carvacrol did not significantly alter t-Akt protein expression; (**D**) Ratio of p-Akt/t-Akt decreased in carvacrol (500 μM) treatment group. * *p* < 0.05, *n* = 6; (**E**) Carvacrol (250 and 500 μM) treatment significantly reduced p-ERK1/2 protein expression. * *p* < 0.05, *n* = 6; (**F**) Carvacrol did not significantly alter t-ERK1/2 protein expression; (**G**) Ratio of p-ERK1/2/t-ERK1/2 decreased in carvacrol (250 and 500 μM) treatment group. * *p* < 0.05, *n* = 6.

Next, we measured whether TRPM7 blocked by carvacrol could regulate both PI3K/Akt and MEK/ERK signaling pathways. As shown in Figure 6A, the representative images of western blotting results indicate weaker p-Akt and p-ERK1/2 bands in carvacrol treatment groups. Densitometry analysis showed that carvacrol (250 and 500 μM) significantly reduced p-Akt protein expression (Figure 6B, carvacrol (250 μM): 84.3% ± 3.1% of control; carvacrol (500 μM): 56.6% ± 4.3% of control, * *p* < 0.05, *n* = 6) and p-ERK1/2 protein expression (Figure 6E, carvacrol (250 μM): 48.9% ± 6.6% of control; carvacrol (500 μM): 65.9% ± 11.6% of control, * *p* < 0.05, *n* = 6). The total Akt and ERK1/2 levels were not significantly different between the control and carvacrol treatment groups (Figure 6 C,F, *p* > 0.05, *n* = 6). Thus, both ratios of p-Akt/t-Akt and p-ERK1/2/t-ERK1/2 decreased in the carvacrol (250 and 500 μM) treatment groups (Figure 6 D,G, * *p* < 0.05, *n* = 6).

### 2.8. Discussion

In this study, we demonstrate that: (1) xyloketal B reduces cell viability, proliferation, and migration of glioblastoma U251 cell lines; (2) xyloketal B downregulates the PI3K/Akt and MEK/ERK signaling pathways; and (3) xyloketal B blocks the TRPM7 current without altering the TRPM7 protein expression in U251 cells. Furthermore, we report that the TRPM7 inhibitor carvacrol can induce effects similar to those of xyloketal B on U251 cells by inhibiting the PI3K/Akt and MER/ERK signaling pathways. We report here, for the first time, the anti-glioblastoma bioactive effects of the marine compound xyloketal B and the underlying signaling pathways, as well as its ability to block the TRPM7 current.

Xyloketal B, obtained from mangrove fungus *Xylaria* sp. (No. 2508), has a novel chemical structure [20], which appealed to other researchers’ interest and was synthesized [32]. Our previous studies reported several bioactive functions of xyloketal B, including protection against endothelial oxidative injury, neuroprotective effects, antioxidant activity, and reducing neonatal hypoxic-ischemic brain injury [21,22,23,24,25,33,34,35]. Further, it has been suggested that xyloketal B could exert multiple pharmacological properties and may be a candidate compound in the treatment of cardiovascular and nervous system diseases. This study explored the effects of xyloketal B on U251 cells, a human glioblastoma cell line. We found that xyloketal B reduced the viability of U251 cells in a dose-dependent manner, at least partly through its inhibitory effects on the proliferation of U251 cells. Furthermore, xyloketal B also inhibited the migration of U251 cells. The intrinsic nature of GBM tumor cells is that they are highly proliferative, migratory, and invasive. Current therapeutic approaches for glioblastoma include surgery, chemotherapy (temozolomide (TMZ), a DNA alkylating agent), and radiation therapy [36]. However, glioblastoma cells have a high resistance to death-inducing stimuli such as radiotherapy and chemotherapy. Thus, it is critical to continuously search for new therapeutic targets and drugs for glioblastoma. The chemical structure of marine compound xyloketal B is distinct from the existing drugs in clinical therapy, hinting that xyloketal B may serve as a novel candidate compound for the treatment of glioblastoma.

Tyrosine kinase expression and subsequent signaling pathway have long been implicated in the pathogenesis of GBM [37]. Amplification of RTKs in GBM leads to an overactivation of the PI3K/Akt signaling pathway and appears in approximately 45% of GBM cases [38]. Preclinical experiments showed that suppression of PI3K/Akt signaling inhibited the growth of glioblastoma [39]. Selective inhibitors of PI3K/Akt signaling pathway have been undergoing clinical trials [40]. Akt, a downstream serine/threonine kinase in the RTKs/PI3K signaling pathway and an appealing target for potential therapy in treating glioblastoma, is up-regulated in phosphor-Akt levels in the majority of GBM tumor samples and cell lines, and causes an enhanced cell proliferation, migration, and invasion [41]. This study demonstrated that xyloketal B decreased p-Akt level in U251 cells, suggesting that downregulation of the PI3K/Akt signaling pathway is involved in anti-proliferation and migration effects of xyloketal B. In terms of the MEK/ERK signaling pathway, it is also constitutively activated by RTKs. RTKs, through a series of adaptor proteins and exchange factors, stimulate Ras activation, which recruits Raf to the plasma membrane, and then phosphorylates and activates MEK, subsequently phosphorylating and activating ERK1/2 [42]. Activation of ERK1/2 regulates both cytosolic proteins and transcription factors involved in cell proliferation, migration, and invasion [42]. Selective inhibitors of the MEK/ERK signaling pathway are currently under clinical trial in GBM therapies [43]. Moreover, there is a crosstalk between the PI3K/Akt and MEK/ERK signaling pathways in maintaining the self-renewal and tumorigenicity of glioblastoma-like stem cells [44], which may contribute to the unsuccessful therapeutic effects of inhibitors in targeting a single signaling pathway [45]. This suggests that a combined blockage of both PI3K/Akt and MEK/ERK pathways would be a rational and effective approach in treating glioblastoma [44]. Besides reducing p-Akt levels, xyloketal B decreased p-ERK1/2 protein expression in U251 cells. These results indicate that xyloketal B downregulates both the PI3K/Akt and MEK/ERK1/2 pathways, contributing to anti-proliferation and migration effects.

Recently, Liu *et al.* reported that knockdown of TRPM7 inhibited proliferation, migration, and invasion of glioma stem cells and revealed that the JAK2/STAT3 and/or Notch signaling pathways were involved in these effects [11]. The function of TRPM7 on glioma stem cell was further confirmed in glioblastoma A172 cells [10]. Thus, TRPM7 is likely a promising target for therapeutic intervention in glioblastoma. There are several lines of evidences suggesting that TRPM7 regulates the PI3K/Akt and MEK/ERK pathways. Silencing TRPM7 decreased the level of p-Akt in OVCA cells and human lung fibroblasts, and also decreased the level of p-ERK1/2 in breast cancer cells [46,47,48]. However, silencing TRPM7 in endothelial cells enhanced cell proliferation, migration, and p-ERK1/2 expression [49,50], suggesting that TRPM7 exerts its function in relation to specific cell types. In this study, we found that xyloketal B blocked TRPM7 currents in HEK-293 cells overexpressing TRPM7. Moreover, we detected TRPM7 protein expression in U251 cells, while xyloketal B did not regulate its protein expression. These data indicate that xyloketal B reduces levels of p-Akt and p-ERK1/2 in U251 cells via blockage of the TRPM7 current. Carvacrol was reported to be an inhibitor of TRPM7 [31], which was also confirmed in our study (data not shown). In this study, similar to xyloketal B, carvacrol was found to reduce cell viability, proliferation, migration, and expression levels of p-Akt and p-ERK1/2 in U251 cells in the parallel experiments. Hence, these results provide evidence that blocking TRPM7 with xyloketal B is a critical step in regulating the PI3K/Akt and MEK/ERK pathways, which are involved in the suppression of cell proliferation and migration of U251 cells. Our study is consistent the previous findings in cancer cells [46,47,48].

The mechanism by which TRPM7 regulates the PI3K/Akt and MEK/ERK pathways remains unclear. Ca^2+^ is a critical second messenger for signal transduction in regulating gene expression, cell proliferation, cell migration, and cell survival, among other activities [51]. In RTK’s signaling pathway, Ca^2+^ interacting with phosphoinositide-specific phospholipase C (PLC), regulates downstream signaling, including PI3K/Akt and MEK/ERK signaling [52]. TRPM7 not only has a very high permeability for both Ca^2+^ and Mg^2+^, but also an α-type serine/threonine protein kinase domain, which has been shown to form a dimer that can autophosphorylate as well as phosphorylate protein substrates [53]. Studies have shown that the C2 domain of several phospholipase C (PLC) isozymes interacts with TRPM7α-type serine/threonine protein kinase domain [54,55]. In our previous study, xyloketal B was shown to regulate calcium entry in endothelial cells and primary cortical neurons [21,25]. Hence, we could speculate that TRPM7 regulates PI3K/Akt and MEK/ERK1/2 signaling through Ca^2+^ entry and interaction with PLC, and thus is involved in the effects of xyloketal B on U251 cells.

In summary, our findings indicate that the effects of marine compound xyloketal B in anti-proliferation and migration of U251 cells are mediated by inhibition of TRPM7 and regulation of the PI3K/Akt and MEK/ERK signaling pathways. Thus, marine compound xyloketal B may be a potential target for drug development for treatment of glioblastoma.

## 3. Experimental Section

### 3.1. Reagents

Anti-TRPM7 (cat #ab85016) was purchased from Abcam (Cambridge, MA, USA). Phosphor-Akt (ser 473,) antibody (p-Akt, cat #9271), Akt antibody (t-Akt, cat #9272), and phospho-p44/42 MAPK antibody (p-ERK1/2, cat #5726) were purchased from Cell Signaling Technology (Danvers, MA, USA). Anti-MAP Kinase ERK1/ERK2 Rabbit pAb (t-ERK1/2, cat #442704) was purchased from Millipore (Billerica, MA, USA). Anti-β-actin was purchased from Sigma-Aldrich (St. Louis, MO, USA). Pierce™ BCA Protein Assay Kit was a product of Pierce Biotechnology (Rockford, IL, USA). All cell culture materials were obtained from Gibco Life Technologies Corporation (Burlington, ON, USA). All other reagents used were purchased from Sigma-Aldrich (St. Louis, MO, USA) unless mentioned otherwise.

### 3.2. Cell Culture

The permanent, well-characterized human glioblastoma cell line U251 was received from the American Type Culture Collection (Manassas, VA, USA) and maintained in Dulbecco’s modified eagle’s medium (DMEM) supplemented with 10% heat-inactivated fetal bovine serum (FBS), 100 U/mL penicillin, and streptomycin in a 37 °C, 5% CO_2_ humidified chamber. HEK-293 cells with stable expression of Flag-murine TRPM7/pCDNA4 were maintained in MEM supplemented with 10% FBS, blasticidin (5 μg/mL), glutamax-1 (2 mM), and zeocin (0.4 mg/mL). HEK-293-Flag-TRPM7 cells were incubated with 1 μg/mL tetracycline to induce TRPM7 overexpression.

### 3.3. Cell Viability and Proliferation

MTT assay was employed to assess cell viability and cell proliferation, as previously described [21]. U251 cells, seeded in 96-well culture plates at a density of 2 × 10^4^ cells/mL, were treated with various concentrations of xyloketal B (31.25–1000 μM) or carvacrol (from 125 μM to 1000 μM) for the described time points (24, 48, or 72 h). MTT reagent (0.5 mg/mL MTT) in completed medium (100 μL) was added to each well and incubated in a CO_2_ incubator for 3 h. Then, the medium was aspirated from each well and 200 μL DMSO was added. The quantity of formazan product of MTT, as measured by absorbance, is directly proportional to the number of living cells. The absorbance was measured in a microplate reader (Syngery H1, Biotek, Winooski, VT, USA) at 490 nm. Cell viability was expressed as a percentage of the control value (0.1% DMSO).

### 3.4. Colony Formation

Glioblastoma U251 cells (300 cells/well) were seeded in six-well plates overnight and subsequently treated with xyloketal B (300 μM) or carvacrol (500 μM) for 24 h. The culture medium was changed at regular time intervals. After seven days of culture, the cells were washed twice with PBS, and fixed with 4% paraformaldehyde for 30 min at room temperature. The colonies were stained with 0.1% crystal violet for 10 min, then washed with water and air-dried. Cell colonies in plate were scanned using CanoScan LiDE 700F and images were captured using a digital camera connected to a phase-contrast Olympus microscope (CKX41, ×10 objectives). The number of colonies containing >50 cells was counted using Image-Pro Plus software (version 1.47V). Data were presented as a percentage of control.

### 3.5. Cell Migration

Wound healing assay was employed to assess cell migration, as described elsewhere [56]. In brief, U251 cells were seeded in six-well culture plates at a density of 5 × 10^4^ cells/mL and grown overnight. Wound gap of the monolayer of cells was created using a 200 μL pipette tip. Then, the cells were treated with xyloketal B (300 μM), carvacrol (500 μM) and vehicle control (0.1% DMSO) at various time points. Cell images of each time point were taken with a digital camera connected to a phase-contrast Olympus microscope (CKX41, ×10 objective). The same visual field was marked and used throughout the experiment. The area of wound gap was measured by Image-Pro Plus software with the wound healing tool. Wound closure (%) = [Gap area (T − T_0_)/Gap area T_0_] × 100% (where T is the treatment time and T_0_ is the time that the wound was induced).

### 3.6. Western Blotting

Western blotting experiments were carried out as previously described [21,57]. Total cell lysates were prepared by scraping cells in RIPA buffer plus proteinase inhibitor cocktails (50 mM Tris, 150 mM NaCl, 1 mM EDTA, 1% Triton X-100, 0.1% SDS, 1% Sodium deoxycholate, 1 mM PMSF, 1 mM Na_3_VO_4_, 1 mM NaF, 1 μg/mL aprotinin, 1 μg/mL leupeptin, 1 μg/mL pepstatin) and centrifuged at 13,000 rpm to pellet the insoluble material. The protein concentrations of cell lysates were determined using was determined with the bicinchoninic acid (BCA) assay method. Equivalent amounts of protein were separated in SDS-PAGE gel and transferred to nitrocellulose membrane (Millipore, Billerica, MA, USA) using a semi-dry transfer method (200 mA per gel, 60 min). The membrane was then blocked with 5% milk in TBS with 0.1% Tween-20 at room temperature for 1 h, and immunoblotted with primary antibodies overnight at 4 °C as follows: anti-TRPM7 (1:1000), anti-p-Akt (1:1000), anti-Akt (1:1000), anti-p-ERK1/2 (1:1000), anti-ERK1/2 (1:1000), and anti-β-actin (1:1000) antibodies, followed by incubation with the corresponding HRP-labeled secondary antibody (Cell Signaling Technology, Danvers, MA, USA, 1:8000) for 1 h at room temperature in conjunction with a chemiluminescence reagent system (PerkinElmer Life Sciences Inc., Boston, MA, USA). Densitometry was carried out using Image-Pro Plus software.

### 3.7. Patch-Clamp Recording

Whole-cell patch-clamp recording was used to analyze TRPM7 currents using an Axopatch 700B (Axon Instruments, Inc., Sunnyvale, CA, USA), as previously described [58]. Briefly, holding membrane potential was held to 0 mV. Voltage ramp (from −100 to +100 mV for 400 ms) protocol was using to recorded TRPM7 currents with an interval of 5 s at 2 kHz and digitized at 5 kHz. Data were acquired using pClamp 9.2 software and analyzed using Clampfit 9.2. All experiments were performed at room temperature. The patch pipette (3–5 megaohms) was made from hematocrit glass using a micropipette puller (Model P-97, Shutter Instrument, Novato, CA, USA). The pipette solution contained (in mM) 145 cesium methanesulfonate, 8 NaCl, 10 EGTA, and 10 HEPES (pH adjusted to 7.2 with CsOH). The bath solution contained (in mM) 140 NaCl, 5 KCl, 2 CaCl_2_, 20 HEPES, and 10 glucose (pH was adjusted to 7.4 with NaOH). The cell was perfused with bath solution before perfusing with xyloketal B (300 μM). When the effects of xyloketal B reached the maximum platform, the cell was reperfused with bath solution to wash out xyloketal B.

### 3.8. Statistical Analysis

Data are presented as the mean ± SEM. One-way ANOVA with subsequent Newman–Keuls test was used to determine the statistical significance for multiple comparisons. Comparison between two groups was analyzed using Student’s *t*-test. All reported *p*-values were two-sided and were considered to be statistically significant at *p* < 0.05.

## 4. Conclusions

Marine compound xyloketal B reduces cell viability, proliferation, and migration through inhibition of TRPM7 and modulation of the PI3K/Akt and MEK/ERK signaling pathways. Thus, marine compound xyloketal B may be a potential target for drug development. Further research, especially *in vivo* study, is essential to explore its value as a potential therapeutic agent for treatment of glioblastoma.
